# Creative reconstruction: a structured literature review of the early empirical research on the COVID-19 crisis and entrepreneurship

**DOI:** 10.1007/s11301-021-00221-0

**Published:** 2021-05-10

**Authors:** Andreas Kuckertz, Leif Brändle

**Affiliations:** Entrepreneurship Research Group, University of Hohenheim, Wollgrasweg 49, 70599 Stuttgart, Germany

**Keywords:** COVID-19, SARS-CoV-2, Coronavirus, Entrepreneurship, Uncertainty, Opportunity, Resilience, Structured literature review, L26

## Abstract

The COVID-19 crisis triggered by the novel coronavirus (SARS-CoV-2) and the infection control measures taken have extended beyond affecting health issues to impact economic activity worldwide. In this structured literature review, the focus is specifically on how the crisis has impacted entrepreneurial activity. The review focuses on the early empirical literature that primarily relied on data collected during the first wave of the pandemic. These empirical results are synthesized in a thematic literature review. The entrepreneurship research on the COVID-19 crisis is marked by three perspectives: the uncertainty perspective, the resilience perspective, and the opportunity perspective. To obtain a complete picture of the effects of the crisis on entrepreneurship, these three perspectives should be considered in combination. We provide implications for future research, policymakers, and entrepreneurs discussing how the interplay of the reviewed perspectives provides paths toward creative reconstruction, that is, the opportunity to move beyond pre-crisis levels of innovation and entrepreneurial action.

## Introduction

Since the beginning of 2020, the world has been experiencing an unprecedented major exogenous shock that has affected society and economies globally to a degree unseen since World War II. The shock was occasioned by the discovery in late 2019 of the severe acute respiratory syndrome coronavirus 2 (SARS-CoV-2) that caused the coronavirus disease 2019 (COVID-19). In addition to a dramatic death toll and pressure on health systems worldwide, the economic effects of combating the virus have also been substantial.

The present paper focuses on the economic actors who engage in entrepreneurial activity. Usually, entrepreneurs who perceive opportunity and establish new organizations to benefit from opportunity (Shane and Venkataraman 2000) are viewed as agents of change who disrupt markets and build the future. In light of the exogenous shock occasioned by the COVID-19 pandemic, entrepreneurs worldwide were forced to confront unexpected change in almost every area of activity, be that changing consumer demand, novel policy measures required to control rates of infection, or investors adjusting their portfolios.

A plethora of academic publications addressing the changing entrepreneurial landscape has attempted to make sense of this situation. However, more often than not and due to the urgency and topicality of the phenomenon, these academic perspectives took the form of editorials, commentaries, and conceptual pieces. While these are certainly helpful contributions, they naturally lack the necessary rigor and evidence to sufficiently assess the impact of the COVID-19 crisis on entrepreneurial phenomena. Available reviews on entrepreneurship and the COVID-19 pandemic tend to focus on specific areas such as sports entrepreneurship (Ratten 2020a) or entrepreneurship education (Ratten 2020b) and thus provide only a fraction of the overall picture. This situation prompted the current structured literature review (Denyer and Tranfield 2009; Fisch and Block 2018; Tranfield et al. 2003) of available *empirical* studies at the interface of COVID-19 and entrepreneurship, thereby going beyond mere informed opinion and focusing on substantial evidence. Consequently, the research question guiding this review is: How does a major exogenous economic shock such as the COVID-19 pandemic affect entrepreneurial activity?

Employing a rigorous and reproducible way of selecting literature seems an effective way to systemize the available evidence. The current research identifies 34 empirical studies that inform the research question. A thematic analysis of this sample suggests that uncertainty, resilience, and entrepreneurial opportunity are particular aspects that should be considered in attempts to understand the effects of the crisis on entrepreneurship. Moreover, these aspects have to be seen as operating in conjunction rather than separately, as a holistic view of the literature makes it possible to define entrepreneurship in times of crisis as creative reconstruction, which goes beyond a simple resilience perspective.

This paper constitutes one of the first general reviews on the Covid-19 pandemic and its consequences on entrepreneurial activities. The findings potentially contribute to three current research streams. First, understanding the mechanics of a specific crisis such as the COVID-19 pandemic adds to the general literature on crisis and entrepreneurship that emerged in the last decade. This research stream has to date primarily focused on the 2008 financial crisis or the effects on entrepreneurship of natural disasters and is likely to benefit from the in-depth discussion of an additional crisis event. Second, with its focus on entrepreneurship, this article aims to complement the economic COVID-19 literature, thereby illustrating an important building block of any broader economic perspective. Given that entrepreneurship can be considered the fundament of economic activity in every free-market economy, any analysis of the economic effects of a crisis would be incomplete without a consideration of entrepreneurship. Finally, the paper suggests opportunity-seeking is the ultimate form of resilience building and thus offers a proposal that contributes to the literature on the enabling factors of entrepreneurial activity. With this opportunity focus, it offers at least a partially positive view on the COVID-19 pandemic and entrepreneurship going beyond the defensive perspectives described previously that primarily concentrate on building resilience simply to restore and preserve the pre-crisis status quo and thereby neglect the potential of building the future.

## Materials and methods

To identify suitable literature addressing the research question in a structured way (Clark et al. 2021), the CIMO (context, intervention, mechanisms, outcomes) framework (Booth et al. 2012) is used to develop search terms that can be used against different databases. Generally, CIMO suggests following its four component steps to narrow a particular search. Given the research question of this paper asking how the exogenous shock of the COVID-19 pandemic has affected entrepreneurial phenomena, the search term is restricted to the first two steps of CIMO, context, and intervention. The final developed search term accordingly reflects entrepreneurial activity as context and the exogenous COVID-19 shock as intervention. The approach ensures a “balance between breadth and depth” (Fisch and Block 2018: 104), as further narrowing the search would have reduced the final sample of studies too much to ensure reasonable analysis.

The search term was applied to three different databases (Scopus, ScienceDirect, and EBSCO (including Business Source Premier and EconLit)) and adjusted following the specific requirements of those databases. Being both Elsevier products, ScienceDirect should be completely covered by the Scopus database; ScienceDirect was nonetheless consulted to correct for potential differences in update cycles of the databases. All databases were queried on January 19, 2021, that is, exactly one year after SARS-COV-2 was first documented in the academic literature (Zhu et al. 2020) thus targeting the early published research in this emerging research stream. The basic Scopus search term (which for other databases was adjusted to match their respective search requirements) was:

(entrepreneu* OR startu* OR start-u* OR ventur*) AND (sars-CoV-2 OR sarsCoV2 OR COVID-19 OR COVID19 OR corona OR coronavirus OR “corona virus”)

**Fig. 1:**
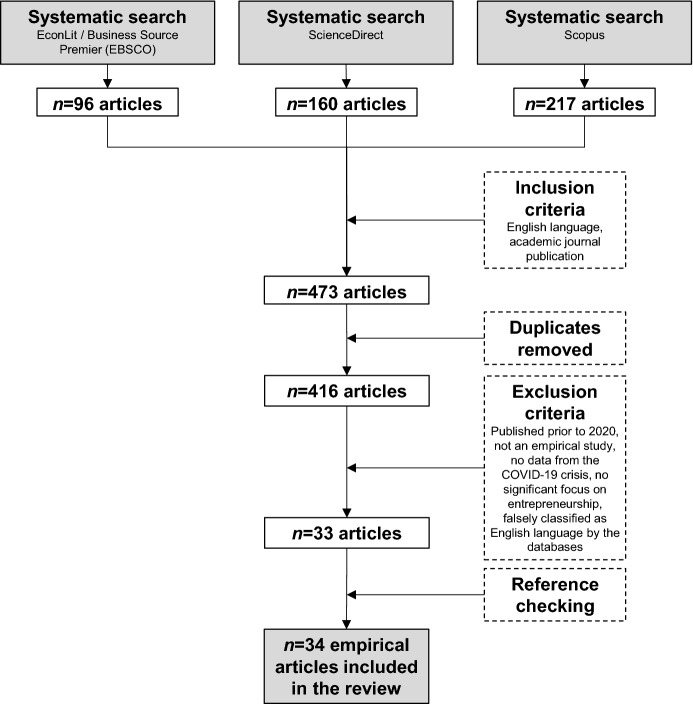
Search funnel of the systematic literature review

The inclusion criteria were academic journal articles published in the English language, thus only including refereed publications and avoiding unrefereed working papers. The search was run over the database fields title, abstract, and keywords. This procedure resulted in an initial sample of *n* = 473 articles. Removing double entries led to *n* = 416 articles. Full-text inspection helped to narrow that sample. Exclusion criteria applied led to the removal of false hits such as articles published prior to 2020 and those not published in English (this applies to articles that, for instance, only provided an English language title and abstract but were written in other languages and therefore produced false hits when querying the databases). Moreover, articles were removed that did not report the results of an empirical study, that did not base their empirical analysis on original data collected throughout the COVID-19 pandemic, and that did not document evidence directly relevant to entrepreneurship. Reference checking (Booth et al. 2012) helped to identify one additional study to be added to the sample. This procedure resulted in *n* = 34 articles relevant to the research question. Figure 1 summarizes the search funnel. Employing a quality cut-off (for instance, in terms of only considering studies published above a certain impact factor threshold, as is common in many published structured literature reviews) seemed inadvisable, as it would potentially have further reduced an already modest sample size.

To analyze the sample, a data extraction sheet was constructed following the recommendations set out by Booth et al. (2012). Table 1 includes an extract of that data extraction sheet and characterizes all articles included in the review. As is typical for an emerging research stream, grounded qualitative research approaches make up a substantial part of the sample, which is roughly evenly divided between qualitative (47.1%) and quantitative approaches (64.7%).^1^ Interestingly, only in a minority of the studies were researchers able to build upon secondary data (21.2%). In the studies, the available data were considered insufficiently informative to make sense of the crisis effects on entrepreneurship, which is why the majority of studies relied on creating original, primary data (76.5%). Table 1 also contains the geographical areas that were investigated by the studies as indicated by the column *data origin*. The studies focus on European economies (50.0% of all studies in the sample), with an additional emphasis on Asia (23.53%) and North America (17.65%). Evidence from South America is rare (5.88%) and the sample contains no information at all about the effects of the COVID-19 pandemic on entrepreneurship in African economies (0.0%). Two studies (5.88% of the sample) adopt a perspective spanning several continents.

**Table 1:** Articles included in the systematic literature review

Paper	Research question	Primary focus	Research paradigm	Data origin	Sample	Method	Key results
Bacq et al. (2020)	Which organizing principles enable rapid and innovative responses to crises?	Opportunity	Primary qualitative	USA	One collaborative online event	Autoethnographic account	Effective organizing principles for online hackathons and idea blitzes
Bandaranaike et al. (2020)	How can an entrepreneurial mindset be induced in students under the conditions of the COVID-19 pandemic?	Resilience	Primary quantitative	Mexico	203 students	Univariate statistical analysis	Reflection helps to develop an entrepreneurial mindset even in online education
Beland et al. (2020)	What are the effects of COVID-19 on entrepreneurs?	Uncertainty	Secondary quantitative	Canada	Canadian Labor Force Survey: 56,000 households and 100,000 individuals	Univariate statistical analysis	A sharp decline in entrepreneurial activity with the first lockdown
Björklund et al. (2020)	How can entrepreneurs experiment with new opportunities and business models in times of crisis?	Opportunity	Primary qualitative	Finland	844 social media posts of 66 ventures in food and beverages; 17 interviews	Thematic analysis; content analysis	Identification of four pathways to expand the entrepreneurial solution space
Block et al. (2021)	What are the factors determining bootstrap financing in a crisis?	Resilience	Primary quantitative	Germany	17,046 entrepreneurial ventures	Logistic regression and negative binomial regression	Individual perceptions of the severity of the crisis for the firm, private consumption, and entrepreneurial experience determine the use of bootstrap finance
Brown and Rocha (2020)	How is the COVID-19 pandemic in China affecting entrepreneurial finance?	Uncertainty	Secondary quantitative	China	13,729 funding transactions	Univariate statistical analysis	Equity funding in Chinese seed-stage ventures drastically declined during the crisis
Brown et al. (2020)	How does the COVID-19 lockdown in the UK affect entrepreneurial finance?	Uncertainty	Secondary quantitative	UK	12,259 funding transactions	Univariate statistical analysis	Equity funding in UK seed-stage ventures declined drastically during the crisis
Cepel et al. (2020)	How do entrepreneurs evaluate pre-pandemic risk and in-pandemic risk?	Uncertainty	Primary quantitative	Czech Republic and Slovakia	1,502 entrepreneurs	Bivariate analysis	Financial risk rises with crisis, risk related to human resources declines
Dumitrasciuc and Turnea (2020)	Which relevant trends for entrepreneurship emerged from the COVID-19 crisis?	Opportunity	Primary quantitative	Romania	117 entrepreneurs	Univariate analysis	Freelancing, digital entrepreneurship, and globalization benefit from the crisis
Ebersberger and Kuckertz (2021)	Which type of innovator is the quickest to react to the COVID-19 crisis?	Opportunity	Secondary quantitative	Global	136 innovations	Network analysis; Poisson QML regression; specification curves	Startups introduce crisis-induced innovations significantly faster than corporates and research institutions
Escamilla-Fajardo et al. (2020)	How has the COVID-19 crisis changed sports entrepreneurship?	Resilience	Primary quantitative	Spain	145 sports clubs	Bivariate analysis, regressions	Risk-taking and innovativeness increase with the crisis, proactiveness remains unchanged; all dimensions of entrepreneurial orientation help to explain service quality
Fairlie (2020)	What are the effects of COVID-19 on entrepreneurial activity?	Uncertainty	Secondary quantitative	USA	130,000 individuals from a current population survey	Univariate statistical analysis, simulation	A sharp decline of entrepreneurial activity with the first lockdown
Farhoud et al. (2021)	How can social enterprise crowdfunding be successful in a crisis?	Resilience	Primary qualitative	UK	1 social enterprise crowdfunding platform	Translational research	7 strategies in three perspectives for social enterprise crowdfunding platforms to effectively serve social enterprises
Fisher et al. (2020)	What are the dynamics in existing organizations targeting entrepreneurial responses to crisis?	Opportunity	Primary qualitative	USA	Organizers of collaborative online event	Autoethnographic account	Entrepreneurial action in existing organizations requires hustle and people giving themselves “permission” to act
Gheorghiu (2020)	How do entrepreneurs perceive government support during the crisis?	Resilience	Primary quantitative	Romania	6,120 entrepreneurs	Univariate analysis	Entrepreneurs appreciate policy initiatives securing liquidity, criticize a lack of transparency of the conditions, and see potential in digitization
Haneberg (2020)	How do responses to crisis affect inter-organizational learning in entrepreneurial ventures?	Resilience	Primary quantitative	Norway	228 knowledge-based entrepreneurial ventures	Cluster analysis, Tobit regression	Firms’ responses can be clustered into “collaborators,” “supporters,” “responders” and “victims,” the first three clusters significantly impact inter-organizational learning
Hernández-Sánchez et al. (2020)	How does the crisis affect entrepreneurial intention?	Resilience	Primary quantitative	Latin America	934 university students	OLS regression	Negative crisis perception reduces entrepreneurial intention, this is mitigated by individual proactiveness, optimism, and whether individual psychological needs are satisfied
Ignat and Constantin (2020)	How does the COVID-19 crisis affect “entrepreneurial spirit?”	Resilience	Secondary quantitative	Romania	15 official economic indicators	Bivariate analysis	Entrepreneurial resilience is found in less developed areas, but more developed areas nonetheless have the greater potential to rebound
Jaim (2020)	What is the gendered experience of female entrepreneurs in the crisis?	Uncertainty	Primary qualitative	Bangladesh	5 entrepreneurial firms	Case studies	Patriarchal structures affect venture closures
Kuckertz et al. (2020)	What adversity face startups during a crisis, what are their coping strategies, and how does policy aim to support them?	Resilience	Primary qualitative and quantitative	Germany/global	16 ecosystem actors (qualitative) and 152 international media reports (quantitative)	Gioia structures/content analysis	Key dynamics model describes startup behavior in the crisis; policy responses to crisis largely neglect innovative startups
Manolova et al. (2020)	How are female entrepreneurs pivoting their business models in the crisis?	Opportunity	Primary qualitative and quantitative	USA	86 female entrepreneurs, 74 female entrepreneurs, two firms	Univariate statistical analysis; case studies	A crisis should be responded to with a discovery-driven approach that simultaneously reduces risks and seizes opportunity
Mirza et al. (2020)	How does COVID-19 affect the behavior of investment funds?	Resilience	Secondary quantitative	Europe	266 capital market, money market, and alternative investment funds	Univariate statistical analysis	Only social entrepreneurship funds are resilient
Nummela et al. (2020)	How has COVID-19 changed the life of cosmopolitan entrepreneurs?	Uncertainty	Primary qualitative	Finland	25 cosmopolitan entrepreneurs	Reports interview excerpts	Lockdowns limit the mobility of cosmopolitan entrepreneurs, challenge their business model but at the same time reduce stress
Prah and Sibiri (2020)	How does the COVID-19 crisis affect migrant entrepreneurs?	Uncertainty	Primary qualitative	China	26 African migrant entrepreneurs	Univariate analysis, interpretative analysis	Crisis identifies structural disadvantages of migrant entrepreneurs
Rizvi et al. (2020)	What is the effect of COVID-19 on the performance and management of different investment funds?	Resilience	Secondary quantitative	Europe	296 capital market, money market, and alternative investment funds	Univariate statistical analysis	Replicates Mirza et al. (2020), extends the timeframe
Ruiz-Rosa et al. (2020)	How does the COVID-19 crisis influence social entrepreneurship intention?	Uncertainty	Primary quantitative	Spain	558 students (324 pre-pandemic, 234 in-pandemic)	Partial least squares	Crisis significantly decreases social entrepreneurship intention
Salamzadeh and Dana (2020)	What are the challenges of startups in the COVID-19 crisis?	Uncertainty	Primary qualitative	Iran	15 startups	Interviews; focus groups	Suggests six primary categories of challenges in the crisis
Saleh (2020)	What effect does the COVID-19 crisis have on the revenues of entrepreneurs?	Uncertainty	Primary qualitative	Kuwait	14 entrepreneurs	Interviews	Changing consumer demand toward entrepreneurial businesses
Santellano (2021)	Do barriers to institutional support also apply to minority entrepreneurs in the crisis?	Resilience	Primary qualitative	USA	5 entrepreneurs	Interviews	Institutional racism puts minority entrepreneurs at a disadvantage
Seidenschnur (2020)	How does the COVID-19 crisis produce or reinterpret social characters?	Uncertainty	Primary qualitative	Germany	156 social media posts	Content analysis with inductive coding	Six social characters emerge, among them the “crisis entrepreneur”
Setthachotsombut and Sua-iam (2020)	What is the relationship between resilience and business performance?	Resilience	Primary qualitative and quantitative	Thailand	400 entrepreneurs plus 24 industry informants	Structural equation modeling	Resilience is a necessary condition of positive business performance effects
Sultan and Sultan (2020)	What challenges do female entrepreneurs face in a developing economy and which enablers benefit them?	Opportunity	Primary qualitative and quantitative	Palestine	260 female entrepreneurs, 15 interviewees	Univariate and bivariate statistical analysis	Crisis causes financial pressure and is answered with innovation
Thorgren and Williams (2020)	What measures are employed by entrepreneurs to react to an external shock?	Resilience	Primary quantitative	Sweden	456 entrepreneurs	Bivariate statistical analysis	Entrepreneurs react to crisis with defensive actions; the option of innovation is almost always ignored
Zhang et al. (2021)	What are the effects of the COVID-19 pandemic on the peer-to-peer accommodation industry?	Opportunity	Primary qualitative	China	9 hosts in the peer-to-peer accommodation industry	Open coding of interview data	COVID-19 causes a shake-out in the accommodation industry with innovative entrepreneurs shaping its future

## Results

**Fig. 2:**
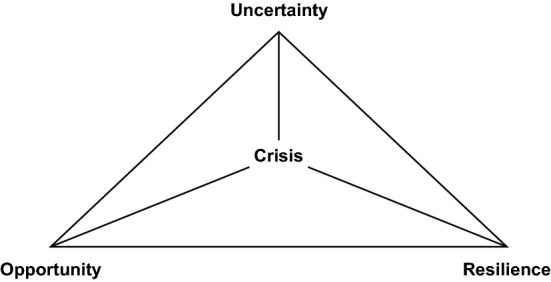
Main topics of the empirical entrepreneurship literature evolving around the COVID-19 crisis

The academic quality control that might feature several rounds of peer review prior to publication affects the results of this literature review and the studies that could be included. Although studies published up to and including January 2021 were considered in the literature review, most studies built their analysis and reasoning predominantly on data on the first wave of the COVID-19 pandemic early in 2020. Although these studies address diverse research questions, a thematic analysis nonetheless suggests three main topics related to the COVID-19 crisis dominate (see Fig. 2). These topics emerged from an initial reading of the sample studies, both authors subsequently assigned all studies based on their respective primary focus independently to one of the topic areas. Additionally, the authors visualized the relationships between empirical concepts and the topic areas in a nomological network. Conflicting classifications and relationships were resolved through discussion within the author team.

First, the crisis caused high levels of uncertainty that went beyond levels that entrepreneurs usually face and the academic literature aims to characterize this uncertainty (35.29% of studies in the sample). Second, resilience is suggested to be a necessary attribute to counter the adverse effects of rising uncertainty, and the academic literature aims to identify those factors that allow entrepreneurs to build such resilience (55.88% of studies in the sample). Third, uncertainty is not seen as a solely negative factor and the changing entrepreneurial landscape also offers new entrepreneurial opportunities (23.3% of studies in the sample). The following paragraphs report how some authors even seem to suggest that a focus on entrepreneurial opportunities is the epitome of resilience, indicating the three main topics are intertwined.

### The uncertainty perspective

The pandemic confronted entrepreneurs with a situation that was not necessarily a black swan event, but which involved having imperfect information to base decisions upon, that is, it was accompanied by high degrees of uncertainty. As a consequence, many otherwise viable business models were challenged (Nummela et al. 2020), in particular by the infection control measures that put much economic activity on hold. When describing this uncertainty, the literature in the sample has primarily focused on the resulting *challenges* for entrepreneurs; reduced *entrepreneurial activity* and *investor* reactions.

Qualitative studies help to illuminate the *challenges* facing entrepreneurs (Kuckertz et al. 2020; Salamzadeh and Dana 2020). Not only do entrepreneurs feel financial pressure, but also pressure in terms of managing their staff and adjusting to changing customer needs. Financial uncertainty is a major factor, as is evident in the findings of surveys of entrepreneurs eliciting their views on the development of the crisis (Cepel et al. 2020). In contrast, however, uncertainty related to human resources seemed to decline—hiring and retaining personnel became easier, as the overall economic uncertainty negatively affected the job market for job seekers as well. Compared to established economic actors, particularly entrepreneurial ventures in the very early stages of development faced additional challenges; not only did they need to a) build and b) operate a growing organization (which already sets them apart from their established counterparts), but they had to engage in crisis management (Salamzadeh and Dana 2020), something they are not usually well prepared for. This uncertainty leads to the threat of non-development, that is, steps envisioned as necessary to establish the enterprise cannot be taken, and sometimes even the very existence of the venture is threatened (Kuckertz et al. 2020).

The uncertainty associated with the COVID-19 pandemic caused a sharp drop in *entrepreneurial activity*. Fairlie (2020) reports entrepreneurial activity in the USA in April 2020 dropped by 22% during the first wave before rebounding over the coming months. Similarly, Beland et al. (2020) report that entrepreneurial activity in Canada dropped by between 10.1 and 14.8% by May 2020. Both studies suggest that minorities, immigrants, less educated individuals, and women were disproportionately adversely affected. Qualitative studies from the Chinese economy (Prah and Sibiri 2020) support this finding and describe how the pressure resulting from infection control measures affected marginalized groups.

*Investors* focused on entrepreneurial ventures reacted to rising uncertainty levels. In the UK, equity funding in seed-stage ventures declined dramatically (Brown et al. 2020). The reason for that decline was at least twofold: equity finance relies strongly on face-to-face interaction, and moreover, investors shied away from new investments and aimed to protect existing portfolios. Similar data are available for the beginning of the pandemic in China, where there too, equity finance for entrepreneurial ventures declined drastically. Those Chinese regions most affected by the COVID-19 pandemic witnessed the greatest reductions in equity finance. The decline in China reported by Brown and Rocha (2020) was three times the magnitude of that seen during the global financial crisis in 2008. The reluctance of investors raises serious concerns as to the innovations that could be realized by entrepreneurial ventures in the coming years, as they are dependent on external funds. Innovation in the near future might therefore be limited (Brown and Rocha 2020). The reluctance on the part of equity investors has led entrepreneurs to increasingly rely on bootstrap financing measures (Block et al. 2021). Beyond that, crowdfunding might be an option offering the prospect of a narrow escape (Farhoud et al. 2021) but has also seen some pressure with some platforms shutting down, at least temporarily.

### The resilience perspective

The negative effects of crisis-induced uncertainty have led researchers to ask what entrepreneurs can do to address it. One answer proposed is to adopt the concept of resilience which describes the capability of an organization to undertake continuous reconstruction (Hamel and Välikangas 2003). The empirical literature on the COVID-19 crisis and entrepreneurship exhibits a threefold approach to resilience. First, it aims to illuminate the *preconditions* of resilience. Second, it illustrates what type of *entrepreneur* is resilient (and which not), and last, it addresses the *policy measures* intended to build resilience among entrepreneurs and their enterprises.

Resilience has positive business performance effects (Ignat and Constantin 2020; Setthachotsombut and Sua-iam 2020) and is a consequence primarily of financial strength and active efforts to manage recovery. Equally, a high level of entrepreneurial orientation (that is, a focus on innovativeness, proactivity, and risk-taking) is empirically suggested to help maintain organizational performance even in times of crisis (Escamilla-Fajardo et al. 2020). Those elements can therefore be said to constitute *preconditions* of resilience.

On the question of what makes a *resilient entrepreneur*, the literature offers more evidence of what undermines an ability to be resilient. Unfortunately, many entrepreneurs take no specific action in response to a crisis, even if they anticipate dramatic effects as Thorgren and Williams (2020) illustrate through Swedish data. If entrepreneurs in this study reacted to the exogeneous COVID-19 shock, they did so primarily by taking defensive action like deferring investments, reducing expenses, reducing labor costs, and renegotiating agreements. Forward-looking options such as a focus on opportunities for innovation were almost completely ignored. This non-resilient behavior in the general population of entrepreneurs intensifies in the case of minority-group entrepreneurs. Fairlie (2020) documents a disproportionate reduction in entrepreneurial activity in the USA among African American and Latino entrepreneurs (and also for female entrepreneurs), which can be ascribed to the fact these groups quite often operate in unfavorable industries such as the retail sector. Therefore, entrepreneurs from minority groups are confronted with an exogenous shock and have to address it from a non-resilient position. For female entrepreneurs (Jaim 2020) who have to operate their business under extremely patriarchal structures (such as are found in some developing economies), those patriarchal structures potentially threaten resilience and exacerbate the effects of a crisis. Interestingly, in a US representative sample, female entrepreneurs with children proved to be more resilient as their activity does not drop as sharply as that of childless female entrepreneurs (Beland et al. 2020). The closure of schools and daycare facilities during lockdowns and increased family obligations have put pressure on this sub-group of female entrepreneurs; however, it seems that having children has led women operating businesses to build more inherent resilience into the enterprise, even before the crisis.

Against this background, policymakers worldwide have aimed to implement *policy measures* that help entrepreneurs quickly build resilience (Kuckertz et al. 2020). These efforts have focused primarily on maintaining sufficiently high levels of liquidity (Gheorghiu 2020; Kuckertz et al. 2020). If such programs are to be conceptualized, entrepreneurs seem to favor suspensions of turnover tax, bank charges, and interest payments, alongside a reduction in the employer contribution to social security (Gheorghiu 2020). Established programs, however, have been criticized in the literature. From an innovation perspective, some authors point to these programs missing a focus on nascent and innovation-driven ventures (Kuckertz et al. 2020). From a justice perspective, other authors highlight that institutional racism leads to some entrepreneurs being unduly excluded from policy support, for instance, Latino entrepreneurs in the USA (Santellano 2021).

### The opportunity perspective

Business models have suddenly been exposed as being no longer viable and the overall uncertainty of the COVID-19 crisis has created a pressure to adapt (Kuckertz et al. 2020). Some authors have looked beyond resilience and suggested innovation can address the challenges facing entrepreneurs (Sultan and Sultan 2020), with some even suggesting that only innovative enterprises will survive the crisis and prosper in the future (Zhang et al. 2021). In other words, this segment of the emerging COVID-19 literature adopts an entrepreneurial opportunity perspective (Shane and Venkataraman 2000) in treating the COVID-19 crisis as an enabler of entrepreneurship (Davidsson 2020; Davidsson et al. 2020). Empirical studies examined for this literature review have focused particularly on three key aspects: *preconditions*, the *character of entrepreneurial opportunity*, and *approaches* to identify and exploit a crisis-induced opportunity.

With regard to *preconditions*, it seems that resilience and opportunity are not necessarily opposing or alternative concepts, but rather that appropriate answers to uncertainty (among them innovative, opportunity-seeking behavior) require a particular degree of resilience (Setthachotsombut and Sua-iam 2020). Infection control measures such as lockdowns have destroyed otherwise viable entrepreneurial opportunities but have at the same time prompted the discovery and creation of new opportunities. For instance, for some entrepreneurs forced inactivity seemed to reduce stress, and forced them to reflect on their values, lives, and entrepreneurial identity. That process in some cases led to the discovery of new fields for entrepreneurial activity (Nummela et al. 2020). A connected theme is visible in the work of Seidenschnur (2020) analyzing the communication in a regional level social media network. The study identified among the general population a type of communicator labeled the “crisis entrepreneur [who] establishes control over the crisis by buffering its economic consequences through creativity” (Seidenschnur 2020: 10). The above research thus suggests creativity and an entrepreneurial mindset are preconditions of a positive response to a crisis. Interestingly, from an entrepreneurship education perspective, it seems that such a mindset can even be inculcated under conditions of physical distancing (Bandaranaike et al. 2020). Moreover, Hernández-Sánchez et al. (2020) employ a student sample to illustrate that traits usually described as conducive to entrepreneurship—such as proactiveness and optimism—help to maintain entrepreneurial intention, a finding that supports the importance of an entrepreneurial mindset in time of crisis. Opportunity-seeking even has positive effects for a group of entrepreneurs who were otherwise observed to be suffering disproportionately from the crisis, namely women. Manolova et al. (2020), for instance, focus on several samples of female entrepreneurs and illustrate that startups led by women adopted pivoting based on new opportunities to deal with uncertainty. In their first sample collected early in the first wave, roughly a quarter of women-led firms reported adjusting the business model, while a later sample reported half of the firms had made such an adjustment.

The above findings raise the question of what exactly constitutes entrepreneurial opportunity, as triggered by the COVID-19 crisis (*the character of the opportunity*). Generally speaking, uncertainty in any crisis follows from novel problems that characterize the crisis and the consequence of such an exogenous shock is that novel and innovative solutions become necessary and possible (Ebersberger and Kuckertz 2021). Empirical studies conducted in the context of the COVID-19 crisis indicate that sectors such as food shrink, whereas entertainment and the gaming industry might benefit (Saleh 2020) along with digitization services for business and bureaucracy (Dumitrasciuc and Turnea 2020; Gheorghiu 2020). In this particular crisis, many entrepreneurial opportunities related to health but Ebersberger and Kuckertz (2021) analyzed a sample of COVID-19 induced innovations, identifying at least nine additional external drivers of relevant structural changes indicating that opportunities extend beyond the health related. Any enterprise experimenting with new business models to address the effects of a crisis would be wise to note that entrepreneurial opportunity comes with new value creation rather than with adjusted or new revenue models that are based on established value creation (Björklund et al. 2020). Remarkably, in terms of opportunities for social and sustainable entrepreneurship, the empirical COVID-19 literature illustrates a clear discrepancy. Social entrepreneurship funds have proved the only resilient asset class producing positive cumulative abnormal returns over the course of the crisis (Mirza et al. 2020; Rizvi et al. 2020) thus undoubtedly suggesting sustainable entrepreneurship and the solution of societal issues constitute opportunities. Nevertheless, entrepreneurs and innovators have almost completely ignored the opportunities around sustainability in their responses to the COVID-19 crisis (Ebersberger and Kuckertz 2021) and Ruiz-Rosa et al. (2020), comparing pre-pandemic and in-pandemic data, report a significantly reduced intention to pursue social entrepreneurship in a student sample.

This literature review also reveals clear *approaches* adopted to exploit opportunities in a time of crisis. The crisis not only affects opportunity per se but also how entrepreneurship is conducted, for instance, enhancing the focus on collaboration (Haneberg 2020). Entrepreneurial ventures in particular do, under certain conditions, seem well-positioned to respond to a crisis. Comparing different types of economic actors, Ebersberger and Kuckertz (2021) identify startups as the type of economic actor quickest to introduce innovations to address the structural changes resulting from the COVID-19 pandemic. The study explains its empirical finding by signposting entrepreneurial ventures’ ability to embrace iterative approaches, effectual logic, and to exploit their small size to look beyond their organizational borders. The value of iterative approaches that forego detailed planning has also been identified in qualitative studies. The finding is associated with the notion of entrepreneurial hustle, which is defined as “an entrepreneur’s urgent, unorthodox actions that are intended to be useful in addressing immediate challenges and opportunities under conditions of uncertainty” (Fisher et al. 2020: 3). Entrepreneurial hustle thus allows for quick responses to a crisis. To illustrate that ability, Bacq et al. (2020) present an autoethnographic account of an extreme case study to identify the organizing principles behind a quick and innovative response to a crisis. Therefore, entrepreneurial-related hustle mitigates the shortcomings of standard procedures that can be too slow to adequately address a crisis (Fisher et al. 2020). Similarly, bricolage—creating the new from the available rather than acquiring additional resources—has been presented as an appropriate answer to the changes caused by an exogenous shock (Kuckertz et al. 2020). A bricolage response to adversity is essentially opportunity-focused, in that it is not defensive but proactive. Björklund and colleagues (2020) convey the positive effects of such iterative, hustling, and bricolage-oriented approaches. Opportunity-seeking through business model experimentation during a pandemic leads to new internal capabilities and even new relational and ecosystem capabilities. Additionally, prosocial experiments build new relational ecosystem capabilities in entrepreneurial ventures. In other words, when the pre-crisis solution is no longer viable, entrepreneurs experiment with new solutions, and particularly with new value creation, which aids their learning. This experimental answer builds capabilities that make entrepreneurial ventures resilient as a consequence of their opportunity-seeking.

**Fig. 3:**
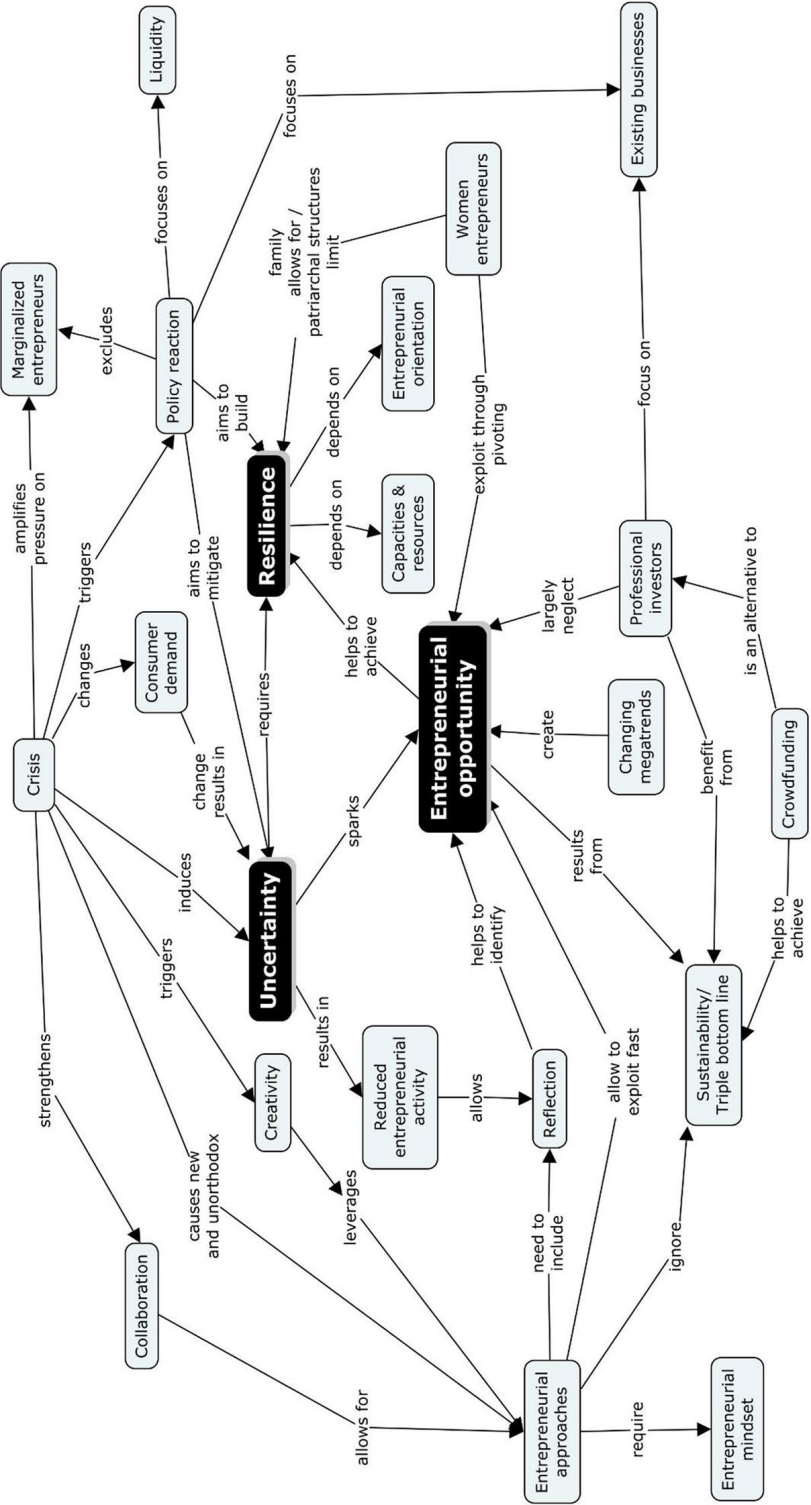
Nomological net (concept map) of empirically suggested relationships

Figure 3 presents the nomological net of the empirically suggested concepts and their relations in a concept map and summarizes the thematic review presented in the preceding paragraphs visually.

## Discussion

### Creative reconstruction as an entrepreneurial answer to crises

This structured literature review illustrates that the COVID-19 pandemic triggered research in three main thematic areas. Researchers primarily focused on describing the uncertainty resulting from the health and economic crisis, shedding light on the concept of resilience that is considered an important resource for firms seeking to thrive in a harsh environment, and most importantly providing a unique, entrepreneurship-research-based perspective on the COVID-19 crisis by focusing on the concept of entrepreneurial opportunities.

Resilience alone is not sufficient to understand entrepreneurial reactions to crises, as it focuses primarily on reconstruction (Hamel and Välikangas 2003), which means the concept is potentially limited because reconstruction might neglect future development through a focus on restoring the pre-crisis status quo. A restoration focus seems inappropriate for entrepreneurial ventures that are naturally oriented towards the future. Although most studies in this structured literature review have adopted an either/or approach by primarily focusing on only one concept, all three—uncertainty, resilience, and opportunity—should be considered in combination.

A (Neo-)Schumpeterian perspective (Hanusch and Pyka 2007) would usually portray entrepreneurship as creative destruction occasioned by entrepreneurs. In light of a crisis such as the COVID-19 pandemic, however, the destruction is exogenously caused, and entrepreneurship becomes *creative reconstruction*, which is useful if enterprises are to move beyond pre-crisis levels when the crisis in question has abated. Such a perspective only becomes feasible if we transcend the resilience perspective and understand opportunity-seeking as the ultimate form of resilience building. In other words, when entrepreneurial activity comes under pressure from a major exogenous shock, entrepreneurship in itself is an integral and essential part of the solution.

A combination of the identified uncertainty, resilience, and opportunity perspectives provides further understanding of the mechanisms of creative reconstruction in times of crisis. The reality is that major entrepreneurial breakthroughs occur during radical environmental changes that represent the “raw material” for entrepreneurs to work with (Davidsson 2020: 323). To this end, there are several implications for future research, for policymakers, and for entrepreneurs highlighting the interplay between the reviewed uncertainty, resilience, and opportunity perspectives. Table 2 summarizes our suggestions in this regard and further illustrates how uncertainty and resilience, uncertainty and opportunity, and resilience and opportunity are related to each other.

**Table 2:** Implications of the interplay of uncertainty, resilience, and opportunity for research, policy, and entrepreneurs

	Implications for future research	Implications for policy	Implications for entrepreneurs
Uncertainty–Resilience	Why do entrepreneurs respond differently to changing environmental uncertainty? Which crises-induced changes are best countered by which entrepreneurial decision logics (e.g., effectuation, bricolage, hustle)? Why do entrepreneurs differ in the way they activate their support systems in a crisis (e.g., networks, policy support)? How are these support systems affected by exogenous shocks?	Increase support for marginalized entrepreneurs experiencing the most adverse consequences of crises Mitigate increased uncertainty in the business environment through factors of resilience beyond mere liquidity (e.g., strengthening the ecosystem)	Build cognitive, relational, and material stock pre-crisis to build an adequate in-crisis response Apply entrepreneurial decision-making logics such as effectuation, bricolage, and hustle to navigate through increased uncertainty
Uncertainty–Opportunity	What is the role of an exogenous shock on technological breakthroughs? What mediates the relationship of an exogenous shock and a technological breakthrough (e.g., enhanced creativity, efficiency, collaboration)?	Support entrepreneurial initiatives countering crises-induced uncertainty early on Strengthen entrepreneurial initiatives to allow for post-crises growth	Remember that while bringing greater uncertainty, crises still provide opportunities to grow Reflect on the new role of the venture in a changed environment Understand that unorthodox entrepreneurial approaches solve crisis-induced problems
Resilience–Opportunity	How do levels of individual resilience correspond to the identification of entrepreneurial opportunities? When do entrepreneurial opportunities emerge as a coping response vs. creating (agency) environmental change? How does crisis experience shape further entrepreneurial path dependence/creation? Will such enterprises pivot more in the future?	Learn from entrepreneurial responses in a crisis by identifying problems only (increasingly) visible during a crisis Move beyond pre-crisis levels via supporting entrepreneurial initiatives that creatively reconstruct the future Prepare for upcoming crises by learning from the efficacy of policy responses and how resilient a policy was to exogenous pressure	Understand that finding ways to adequately respond to a crisis involves the possibility of identifying new entrepreneurial opportunities Make do with what is at hand during crises as this might lead to enhanced focus on value creation Invest effort in overcoming crisis-induced constraints and adversity to trigger learning that instills post-crisis resilience and drives growth beyond pre-crisis levels

### Implications for research

The reviewed entrepreneurship studies illustrate remarkable environmental changes in entrepreneurs’ business landscapes as a consequence of the Covid-19 pandemic. Such environmental changes involve increased uncertainty and shed light on entrepreneurs’ resilience in times of crisis. Nevertheless, future research seeking to improve understanding of a more general relationship between disruptive events and entrepreneurs’ responses might benefit from differentiating between different types of events including the scope of the impact on individual entrepreneurs. For the first operation, Bendell et al. (2020) suggest broadly differentiating between disruptive events such as *crises* (e.g., airplane crash), *disasters* (e.g., hurricane), and *looming mega-catastrophes* (e.g., climate change) based on the duration, severity and impact of the event. Accordingly, further research might look into how entrepreneurial responses differ according to the type of the disruptive event. Entrepreneurs’ resilience, then, also depends on their specific positions prior to the crisis and the scope of the impact on the resources available to them during the crisis. For instance, several studies in this review indicate that minority entrepreneurs are hit especially hard by the crisis (Beland et al. 2020; Fairlie 2020; Prah and Sibiri 2020). By further carving out the mechanisms responsible for the unequal impact of a crisis such as the Covid-19 pandemic (Munir 2020) such as the *liability of poorness* (Morris 2020) on the efficacy of entrepreneurial responses (such as entrepreneurial bricolage and hustle), future research might increase our understanding of how resilience in the face of uncertainty differs depending on the backgrounds of entrepreneurs and the context in which they operate.

A differentiated perspective on the *environmental change–entrepreneurial response* relationship also offers a clear view of how entrepreneurial opportunities emerge from crises (see also, Shepherd 2020). While several reviewed studies indicate that increasing environmental pressure might be a factor leading to the identification of entrepreneurial opportunities, the mediating mechanisms remain largely unclear. For instance, are technological breakthroughs during crises more likely due to enhanced collaboration and efficiency? Startups such as Biontech SE driving the Covid-19 vaccine development in an accelerated rolling review process and adopting large-scale cooperation with established producers provide anecdotal evidence for such propositions. Entrepreneurial opportunities that entail solutions to crises might be characterized by increased collaboration efforts (i.e., including governments and private actors) and efficiency (e.g., time to regulatory approval of a drug or vaccine and time to market). While the results of the literature review indicate that creativity is helpful to successfully respond to crises like the Covid-19 pandemic, future research might investigate how radical environmental changes increase the development of innovative ideas (such as solutions to a crisis, see, Bacq et al. 2020) due to enhanced creativity.

Another promising path for research involves asking how entrepreneurial opportunities emerge from entrepreneurs’ attempts to withstand the adverse effects of environmental changes brought about by crises. The reviewed literature indicates that entrepreneurs’ responses to crises include creatively adjusting business models to reflect the current situation and creating solutions despite exacerbated resource constraints; however, future research might examine whether such responses might not only restore entrepreneurs’ ability to survive in an altered market environment but drive the identification of overlooked entrepreneurial opportunities and produce results exceeding pre-crisis ones. That said, the experience of mastering extraordinary resource constraints and market uncertainty might create a new and irreversible path for those ventures that survive crises such as the Covid-19 pandemic. Creative reconstruction via entrepreneurial action during a crisis would, then, not only benefit economies in that they might emerge stronger by surpassing pre-crisis levels of innovation but also reveal opportunities to accelerate learning toward organizational renewal.

### Implications for policy and entrepreneurs

The reviewed literature indicates that adverse effects in a crisis such as the Covid-19 pandemic are distributed unequally between entrepreneurs and their ventures. Accordingly, policy implications include increasing support for marginalized entrepreneurs during times of crisis. Several studies document the exacerbated effects on female entrepreneurs implying the need for additional policy support (Fairlie 2020; Jaim 2020). However, other studies in the review also highlight the exemplary response of female entrepreneurs (Beland et al. 2020) which indicates a pre-crisis resilience to uncertain and unequal environments. Recommendations for entrepreneurs are, therefore, to either rely on imprinted decision-making logics such as bricolage, effectuation, and hustle or that they apply such heuristics for the first time during a crisis. In other words, building cognitive, relational, and material stock pre-crisis will improve the response in the midst of a crisis (Kuckertz et al. 2020). Therefore, policy support should not only aim to tackle funding gaps for new ventures (as in the financial crisis, see Block and Sandner 2009) but additionally strengthen entrepreneurs’ broader support systems such as the entrepreneurial ecosystem before and during a crisis (e.g., see Ratten 2020c).

While the reviewed literature indicates that crises such as the Covid-19 pandemic increase environmental pressure on entrepreneurs, it also illustrates opportunities that arise specifically due to these environmental changes. The implications for entrepreneurs navigating through such a crisis include the recommendation they undertake a thorough reflection of their venture’s potential role in solving problems caused by the crisis and shaping a post-crisis future. That recommendation flows from the property of a crisis such as the Covid-19 pandemic of disrupting the established playing field in ways that open opportunities for new ventures (Zahra 2021). Unorthodox approaches to solving problems during a crisis and shaping the path of post-crisis recovery via bold entrepreneurial action might pay off for new ventures in the long run. However, policy support implemented early and on a large scale fosters the competition between economic actors to contribute to innovative solutions to crisis-induced problems, which might boost entrepreneurs’ endeavors to effect creative reconstruction. Policy can be applied to accelerate the disruptive process of entrepreneurial actors pivoting toward emerging entrepreneurial opportunities by removing barriers and increasing incentives to contribute to developing post-crises equilibria.

Finally, the reviewed literature indicates that crises such as the Covid-19 pandemic provide opportunities for societal, organizational, and individual learning. For instance, a venture’s enforced pivot during crisis toward activities that generate the most value (e.g., solving problems related to the crisis) might assist in identifying a viable business model that works beyond a time of crisis and enables the venture to surpass its pre-crisis performance. Such learning through navigating crises might also instill improved post-crisis resilience as entrepreneurs and their ventures can draw on such experiences in similar situations in the future. Since crises starkly reveal systemic problems that might have been hidden in times of growth (e.g., Munir 2020), policymakers and entrepreneurs alike should pay attention to working to alleviate such problems to creatively reconstruct a better and more resilient future.

## Conclusion

The COVID-19 pandemic, and the entrepreneurship studies addressing it, provide a unique opportunity to convey how a new research stream emerges, albeit this research stream is part of the larger discussion on entrepreneurship and crisis that has been ongoing for some time (see Doern et al. (2019) for a recent overview). Moreover, although the real-life effects of the crisis are certainly dramatic and are likely to be the defining moment of a generation (Gates 2020), in terms of research, we are far away from witnessing a paradigmatic shift (Kuhn 1962). Instead, key concepts of entrepreneurship research such as the opportunity perspective have proved both fruitful and to exhibit remarkable explanatory power.

The early literature on the effects of COVID-19 on entrepreneurship could, however, be criticized for a lack of theoretical rigor and a focus on small, swiftly assembled, and quite often qualitative samples. This was perhaps inevitable owing to the peculiarities of a crisis that required a rapid response. Future research should not restrict its scope to merely closing apparent gaps in the literature, such as answering the question of which crisis response constituted a viable entrepreneurial response, a question that could only be answered with the benefit of hindsight. Equally, a focus on theoretically grounded research questions able to meet the criteria of delivering rigor, and reliable, representative data seems desirable. Admittedly, such research would only marginally affect how the current crisis is managed both individually and organizationally but would be extremely useful in supporting future generations’ attempts to deal with their own crises.

## Data Availability

Available upon request.

## Footnotes

1 Note percentages here and in the following do not necessarily add up to 100 which is due to some studies employing mixed-method designs that can therefore be assigned simultaneously to more than one category, or some studies reporting data from more than one economy.
